# Comparison of Methods for Visual Field Progression in Eyes With Central Visual Field Defects

**DOI:** 10.1167/tvst.14.11.3

**Published:** 2025-11-06

**Authors:** Takashi Nishida, Robert N. Weinreb, Evan Walker, Christopher A. Girkin, Massimo A. Fazio, Jeffrey M. Liebmann, Sasan Moghimi

**Affiliations:** 1 Hamilton Glaucoma Center, Shiley Eye Institute, and the Viterbi Family Department of Ophthalmology, University of California San Diego, La Jolla, CA, USA; 2 Bernard and Shirlee Brown Glaucoma Research Laboratory, Harkness Eye Institute, Columbia University Medical Center, New York, NY, USA; 3 Department of Ophthalmology and Vision Sciences, Heersink School of Medicine, The University of Alabama at Birmingham, Birmingham, AL, USA

**Keywords:** central visual field, progression, glaucoma

## Abstract

**Purpose:**

To investigate the agreement of various criteria for visual field (VF) progression in eyes with central VF defects, and to evaluate their performance in simulation datasets with and without age-related and glaucomatous change.

**Methods:**

A total of 282 eyes of 197 primary open-angle glaucoma patients with 10-2 central VF defect at baseline with two or more years’ follow-up and five or more visits for both 10-2 and 24-2 VF were included. Various progression detection methods were used: 10-2 clustered pointwise linear regression (PLR), 10-2 VF mean deviation (MD), 24-2 central VF mean total deviation (MTD), 24-2 VF MD, 24-2 PLR, guided progression analysis, Advanced Glaucoma Intervention Study, and Collaborative Initial Glaucoma Treatment Study scores. Progression was defined as a binary outcome at the final visit: ≤−0.7 dB/year for 10-2 VF MD and ≤−0.5 or ≤−1.0 dB/year for 24-2 central VF MTD. Pairwise agreements were evaluated using Cohen's kappa. To further assess the detection performance under controlled conditions, two simulation datasets were constructed: one incorporating realistic progression and another with no true change. The *t*-statistics from ordinary least squares regression were used to compute receiver operating characteristic curves and normalized partial area under the curves.

**Results:**

Central progression was more frequently detected with 10-2 VF: 10-2 VF MD (35.1%) and 10-2 clustered PLR (20.6%) versus 24-2 central VF MTD at ≤−0.5 dB/y (17.7%) and ≤−1.0 dB/y (3.2%). Global progression was observed in 17.7% to 30.5%. The agreement among methods ranged from 67.0% to 85.1%, with kappas values of 0.11-0.25 between 10-2 and 24-2 MTD methods and 0.22–0.54 between 10-2 and 24-2 methods. Simulation analyses confirmed that 10-2 VF MD had the highest partial AUC across specificity levels.

**Conclusions:**

Agreement among methods for central VF progression monitoring is low to moderate. Concordance between 24-2 and 10-2 VF methods is variable, with 10-2 detecting a higher proportion of central progression.

**Translational Relevance:**

Incorporating 10-2 VF testing alongside 24-2 is essential, as relying solely on 24-2 VF may underestimate central VF progression.

## Introduction

Glaucoma, a progressive optic neuropathy, is characterized by changes in the optic nerve head, retinal nerve fiber layer, and visual field (VF).^1^ Monitoring these changes, particularly in patients with central visual field defects (CVFD), is critical for effective disease management and prevention of significant visual impairment.^2^ Central visual field is especially important as it directly impacts quality of life by affecting daily activities such as reading and face recognition.^3^^–^^5^

In clinical practice, VF progression is typically monitored using trend analysis methods that evaluate the rate of change in VF parameters, such as mean deviation (MD) or threshold sensitivity at specific test locations, as well as event detection methods like guided progression analysis (GPA).^6^^,^^7^ In clinical trials, various methodologies are used to evaluate VF progression, including the Advanced Glaucoma Intervention Study (AGIS) criteria,^8^ the Collaborative Initial Glaucoma Treatment Study (CIGTS) scoring system,^9^ and pointwise linear regression (PLR).^10^^–^^13^

The challenge of detecting VF progression lies in the variability of test results, often referred to as *long-term fluctuation*.^14^ This variability can obscure true disease progression, complicating clinical decision-making. Previous studies have highlighted the low concordance among different techniques for detecting VF progression. For instance, Vesti et al.^15^ compared AGIS, CIGTS, glaucoma change probability analysis, and PLR 24-2 methods, finding that the overall agreement between methods was between 50% and 63%, depending on the variability. This study demonstrated that different methods could produce significantly different progression detection rates. Similarly, Rabiolo et al.^16^ compared the performance of nine 24-2 VF progression detection methods, including qualitative clinical evaluation, GPA, MD and VFI rate, AGIS and CIGTS scoring systems, PLR, permutation analyses of PLR, and glaucoma rate index, and reported considerable variability of progression rates, indicating the need for concordance and reliability in assessment methods.

The lack of consensus on the best VF test and method for monitoring VF progression, particularly in patients with CVFD,^15^^–^^17^ underscores the necessity for further research. This study aims to investigate the agreement of various criteria for VF progression in eyes with central VF defects by evaluating the agreement between 10-2 and 24-2 VF tests and multiple methods for detecting VF progression. By comparing the performance of these methods, we seek to inform clinical practice and improve the precision of VF progression monitoring.

## Material and Methods

### Participants

This retrospective cohort study included primary open-angle glaucoma patients enrolled in the Diagnostic Innovations in Glaucoma Study and African Descent and Glaucoma Evaluation Study, conducted at the Hamilton Glaucoma Center at the University of California, San Diego, Columbia University (previously at the New York Eye and Ear Infirmary), and the University of Alabama at Birmingham. The study adhered to the tenets of the Declaration of Helsinki and the Health Insurance Portability and Accountability Act, with Institutional Review Board approval obtained at each site. Written informed consent was obtained from all participants.

**Figure 1.**
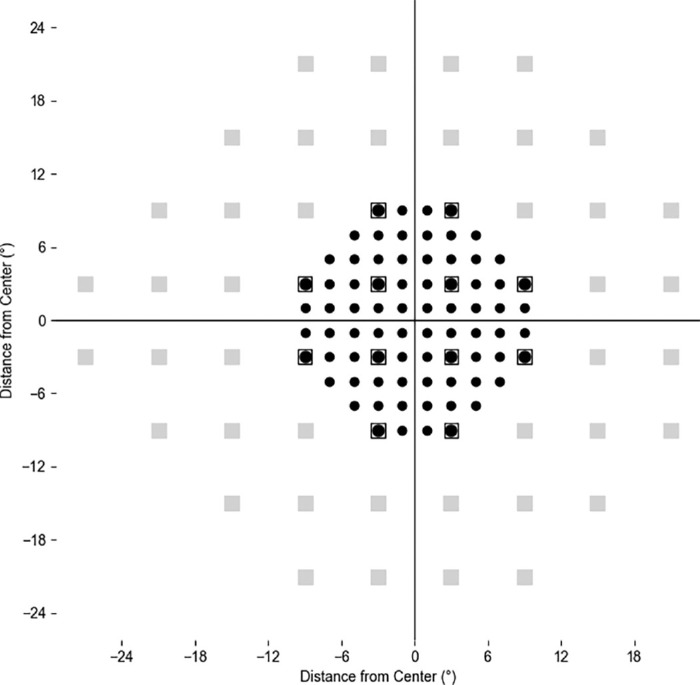
Plot showing the locations of the 10-2 and 24-2 visual field test grids, with *squares* representing the 24-2 test points and *filled circles* representing the 10-2 test points. The 12 central locations, where both test grids overlap, are depicted with *filled circles* inside *unfilled squares* to indicate shared test points. *Light gray squares* represent additional 24-2 test points beyond 10°. Plot is shown for a right eye.

Patients were included if they had a baseline 10-2 CVFD, with at least two years of follow-up and a minimum of five visits for both 10-2 and 24-2 VF testing. A CVFD using 10-2 VF was defined using the following cluster criteria. Specifically, 10-2 hemifields were classified as abnormal if there was a cluster of three contiguous points with probabilities of <5%, with the additional requirement of at least one of the three test points having a probability <1% or two of the three test points having probabilities of <2% within a hemifield on either total deviation or pattern deviation plots.^18^ The plot showing the locations of 10-2 and 24-2 visual field test is provided in Figure 1.

Primary open-angle glaucoma was defined based on repeatable (at least two consecutive) abnormal VF test results and evidence of glaucomatous optic neuropathy. This was defined by excavation, focal thinning, or notching of the neuroretinal rim, or localized or diffuse retinal nerve fiber layer atrophy, as determined from optic disc photographs. Visual field tests were performed using the Humphrey Field Analyzer (Carl Zeiss Meditec, Inc., Dublin, CA, USA), and unreliable tests (>33% fixation losses, >33% false-negative, or >15% false-positive) were excluded. Furthermore, based on standardized assessment by the UC San Diego Visual Field Assessment CenTer (VisFACT),^19^ VF results were excluded if the following artifacts were present: (1) rim artifacts (non-repeatable loss around the VF edge) and eyelid artifacts (non-repeatable loss in the superior VF locations), (2) inattention (elevated false-negatives, generally depressed, or patchy field) or fatigue effects (cloverleaf pattern, normal center with defective edges), and (3) VF damage from diseases other than glaucoma (e.g., pituitary lesions, demyelinating diseases).

Inclusion criteria were (1) age >18 years; (2) open angles on gonioscopy; and (3) best-corrected visual acuity of 20/40 or better at study entry. Exclusion criteria were (1) history of trauma or intraocular surgery (except uncomplicated cataract or glaucoma surgery); (2) coexisting retinal disease, uveitis, or non-glaucomatous optic neuropathy; (3) axial length ≥27 mm; and (4) significant cognitive impairment, Parkinson's disease, Alzheimer's disease, dementia, or history of stroke.

### Visual Field Progression Evaluation

The 10-2 and 24-2 VF progression was assessed using multiple methods (Table 1). Progression was determined as a binary classification at the final visit for each method. To improve clarity, statistics derived from 24-2 and 10-2 VF grids are annotated with subscripts (e.g., MD_10_, MD_24_, PLR_24_) to indicate the corresponding test grid.

**Table 1.** Criteria for Visual Field Progression

		Number of Eyes (%)		
	Criteria	Without Simulation	With Simulation	Assume 0 dB/y for Age-Related Change With Simulation
Methods for 10-2				
10-2 clustered PLR (PLR_10_)	3 test locations having a significant regression slope (*P* < 0.01) of ≤−1 dB/y located in the 10-2 VF sector	20.6%	21.6%	0.00%
10-2 VF MD (MD_10_)	Significant regression slope (*P* < 0.05) of ≤−0.7 dB/y*	35.1%	18.5%	0.01%
Methods for central 24-2				
24-2 central VF MTD (MTD_24_)	Central mean total deviation values of the 12 test locations within the central 10 degrees region of the 24-2: slope of ≤−0.5 dB and ≤−1.0 dB	≤−0.5 dB/y: 17.7%	≤−0.5 dB/y: 13.9%	≤−0.5 dB/y: 0.02%
		≤−1.0 dB/y: 3.2%	≤−1.0 dB/y: 2.0%	≤−1.0 dB/y: 0.00%
Methods for global 24-2				
24-2 VF MD (MD_24_)	Significant regression slope (*P* < 0.05) of ≤−0.5 dB/y	19.5%	33.7%	0.07%
24-2 PLR (PLR_24_)	3 test locations having a significant regression slope (*P* < 0.01) of ≤−1 dB/y	20.6%	35.6%	0.05%
24-2 GPA	Threshold deviation outside the test–retest variability boundaries from the baseline threshold deviation in ≥3 locations maintained in 3 consecutive VFs	30.5%	N/A	N/A
AGIS	Score increased of ≥4 points compared to the baseline test, and maintained in at least 2 consecutive examinations	17.7%	14.3%	1.0%
CIGTS	A worsening of ≥3 points compared with the average of the first 2 tests, and maintained in at least 2 consecutive examinations	27.0%	4.9%	0.00%

* Because there is no established rate of 10-2 VF MD change, the cutoff value with the highest accuracy is calculated from the clustered PLR and MD rate of change (Supplementary Fig. S2).

The 10-2 VFs were assessed by (1) 10-2 clustered PLR (PLR_10_): evaluates each VF location individually, with progression defined by a significant negative slope (*P* < 0.01) of at least −1.0 dB/year in three or more locations in the sector. These three test locations do not need to be adjacent within the sector (See Supplementary Fig. S1)^12^; (2) 10-2 VF MD (MD_10_): evaluates VF MD slope. Because there is no established rate of 10-2 VF MD change, the cutoff value with the highest accuracy is calculated from the 10-2 clustered PLR and 10-2 VF MD rate of change (See Supplementary Fig. S2).

The 24-2 VFs were assessed by (1) 24-2 central VF mean total deviation (MTD_24_), calculated as the mean of the total deviation values from the 12 test locations within the central 10-degree region of the 24-2 test, with thresholds set at ≤−0.5 dB/year and ≤−1.0 dB/year for progression.^20^ MTD is an unweighted measure of the average threshold; (2) 24-2 VF MD (MD_24_): evaluates VF MD slope, significant regression slope (*P* < 0.05) of ≤−0.5 dB/y.^21^ MD is a weighted average of total deviation values, giving more weight to stable and important center and less to variable periphery; (3) 24-2 PLR (PLR_24_): similar to 10-2 PLR but applied to the 24-2 test, with three test locations having a significant regression slope (*P* < 0.01) of ≤−1 dB/y^13^; (4) 24-2 GPA: uses pattern deviation values compared to baseline exams, and defining significant progression by the deterioration of three or more locations on three or more consecutive exams (likely progression)^6^; (5) AGIS scoring: based on the number, depth, and distribution of 24-2 depressed VF locations, with progression defined as ≥4 points increase sustained in at least two consecutive VFs compared to the baseline test^8^; (6) CIGTS scoring: similar to AGIS but with progression defined as a three-point increase sustained in at least three consecutive 24-2 VFs. CIGTS scoring: similar to AGIS but with progression defined as three or more points increase sustained in at least two consecutive VFs.^9^

These metrics have been used to assess VF progression, with their strengths and limitations demonstrated in prior research.^7^ Our study compares the established criteria to further evaluate their clinical utility. Glaucoma disease severity was classified as early (24-2 VF MD >–6 dB), or moderate-to-advanced (MD≤–6 dB) at the baseline.

### Simulation

To evaluate VF progression detection methods in a specificity-controlled framework, we constructed two simulation datasets derived from the same original set of glaucomatous eyes. The first dataset simulated patient-specific progression patterns by adding measurement variability estimated from residual distributions to each eye's original regression-based slope. Following the method of Wu et al.,^22^ we created the simulation into two components: a sensitivity template and a noise template. The sensitivity template was derived from pointwise sigmoid regression models fitted to the longitudinal threshold sensitivities of each eye. These nonlinear models capture the characteristic trajectory of VF decline, with parameters representing the onset, rate, and asymptotic behavior of loss. For locations already at or near floor, the fitted values were fixed at 0 dB. To capture measurement variability, we obtained residuals by subtracting the fitted sensitivities from the observed values at each location and visit. These residuals were binned by the fitted sensitivity (rounded to the nearest 1 dB) to form empirical probability distribution functions. A noise template was then constructed by converting each test's residual pattern into a joint probability map. The resulting noise template retained spatial correlations between test locations. The second dataset assumed no true functional change (i.e., slope = 0 dB/year) at all test locations. It was constructed using the same baseline values from the original eyes, but without any longitudinal trend; instead, only the noise template was added to each series. This formed a null dataset where any apparent change was due solely to variability. In both datasets, test sequences were reconstructed by combining the sensitivity and noise templates. At each time point, a fitted sensitivity value was sampled from the sigmoid model and then perturbed by drawing from the appropriate residual distribution. A total of 100 replications per eye were created, each with a different realization of measurement noise.

All eyes in both datasets were initially evaluated using predefined rule-based progression criteria (Table 1), combining thresholds for statistical significance (e.g., *P* < 0.05) and minimum slope magnitude (e.g., ≤−1.0 dB/year) for trend-based criteria. These thresholds were selected to suppress false-positives, particularly those arising from age-related change,^23^^–^^25^ which can yield negative slopes even in healthy eyes. Although the criteria were highly specific by design, they proved overly stringent in practice, and very few eyes met progression definitions in the 0 dB/year dataset, indicating that categorical progression definitions may have reduced sensitivity when applied to stable VF trajectories.

To address this, the *t*-statistics were calculated from ordinary least squares regression as a continuous indicator of VF change. For each eye, *t*-statistics were computed based on three VF summary measures including MD_10_, MD_24_, and MTD_24_. These *t*-values capture the standardized rate of change (slope divided by its standard error), enabling comparison across eyes with differing noise levels. Receiver operating characteristic (ROC) curves were constructed using these *t*-statistics, with the two simulation datasets serving as empirical distributions of signal-present and signal-absent conditions. Unlike standard ROC analyses, where ground truth is fixed by known labels or thresholds, our approach used the continuous t-statistic both as a predictor and as the basis for defining operating thresholds. Sensitivity and specificity were computed across all possible t-statistic values, yielding performance curves that did not depend on arbitrary cutoffs. We computed the area under the ROC curve (AUC) and also calculated normalized partial AUCs (pAUCs) restricted to false positive rates (FPR) of 0.05, 0.10, and 0.15. Each pAUC was divided by the theoretical maximum under that FPR. Bootstrapped confidence intervals (1000 iterations) were calculated for AUCs and pAUCs, and paired comparisons were used to estimate *P* values across VF summary methods.

### Statistical Analysis

Patient and eye characteristics data were presented as mean (95% confidence interval [CI]) for continuous variables and count (%) for categorical variables. Pairwise agreements among the methods were measured using Cohen's kappa. Because each progression criterion provided binary outcomes (progression or no progression), Cohen's kappa and raw percent agreement were calculated for all 2 × 2 pairwise comparisons.^26^ This analysis was repeated as a stratified analysis based on the baseline severity of glaucoma (i.e., early or moderate-to-advanced stages). UpSet plots were used to visualize the intersections of progression detection across methods. This plot was used to highlight the overlap and unique patterns in progression detection among the different methods, showing how many eyes were identified by each method and their combinations. In addition to the standard intersection-based analysis, UpSet plots in Union mode were generated to provide a broader perspective on progression detection by capturing all eyes identified as progressing by at least one method, thereby highlighting the cumulative detection across different criteria. To compare progression classifications between the MD_10_ and MTD_24_ (<0.5 dB/y), a Venn diagram was created to illustrate the overlap between eyes identified as progressing by the two methods. Linear mixed models were employed to estimate the average rate of change in VF MD over time, using a linear function of time. Participant-specific and eye-specific deviations from this average rate were introduced by random slopes. The model accounts for the varying rates of MD loss in different eyes and accommodates correlations between both eyes of the same individual. For the continuous variables (including MD_24_ slope, MTD_24_ slope, and MD_10_ slope), scatter matrices were created to evaluate the degree of agreement among these indices. Pearson correlation coefficients were calculated to assess the association among these different indexes of VF progression. Statistical analyses were performed using Stata version 16.0 (StataCorp, College Station, TX, USA) and python 3.11.1 (Python Software Cooperation, Wilmington, DE, USA). *P* value was not adjusted for multiple comparisons because of the exploratory nature of the analysis.

## Results

A total of 2766 24-2 VFs and 2797 10-2 VFs from 282 (190 early, 92 moderate-to-advanced) eyes of 197 patients were analyzed. Mean rate of MD_24_ slope was −0.35 (95% CI, −0.35 to −0.28) dB/y over 9.8 (95% CI, 9.3 to 10.3) years, and mean rate of MD_10_ slope was −0.33 (95% CI, −0.41 to −0.26) dB/y over 9.7 (95% CI, 9.2 to 10.2) years. Each patient underwent a mean of 9.8 (95% CI, 9.3 to 10.3) for 24-2 and 9.7 (95% CI, 9.2 to 10.2) for 10-2 VF tests. Baseline mean (95% CI) MD_24_, MD_10_, AGIS score, CIGTS score were −5.6 (−6.1 to −5.0) dB, −5.0 (−5.7 to −4.4) dB, 4.4 (3.9 to 4.9), and 6.6 (6.0 to 7.2), respectively. Demographics and baseline clinical characteristics of the subjects are presented in Table 2.

**Table 2.** Demographic and Baseline Clinical Characteristics of the Participants

Characteristic	Mean (95% CI)
By Subject (No)	197
Age (years)	68.9 (67.4–70.3)
Sex	
Female	100
Male	97
Race	
African American	61
Non-African American	136
By Eye (No)	282
Axial length (mm)	24.2 (24.1–24.4)
CCT (µm)	537.1 (531.7–542.5)
Mean IOP during follow-up (mm Hg)	13.6 (13.1–14.0)
Disease Severity by baseline 24-2 VF MD	
Early glaucoma, Eye No (%)	190 (67.4%)
Moderate-to-advanced glaucoma, Eye No (%)	92 (32.6%)
Baseline 24-2 VF MD (dB)	−5.6 (−6.1 to −5.0)
24-2 VF MD slope (dB/y)	−0.35 (−0.42 to −0.28)
Baseline 24-2 VF central MTD (dB)	−2.7 (−3.1 to −2.4)
24-2 central VF MTD slope (dB/y)	−0.21 (−0.27 to −0.15)
Baseline 10-2 VF MD (dB)	−5.0 (−5.7 to −4.4)
10-2 VF MD slope (dB/y)	−0.33 (−0.41 to −0.26)
Follow-up period for 24-2 VF (years)	6.2 (5.9–6.5)
Follow-up period for 10-2 VF (years)	6.5 (6.2–6.8)
VF follow-up visits for 24-2	9.8 (9.3–10.3)
VF follow-up visits for 10-2	9.7 (9.2–10.2)
Baseline AGIS score	4.4 (3.9–4.9)
Baseline CIGTS score	6.6 (6.0–7.2)

CCT, corneal central thickness; IOP, intraocular pressure.

**Figure 2.**
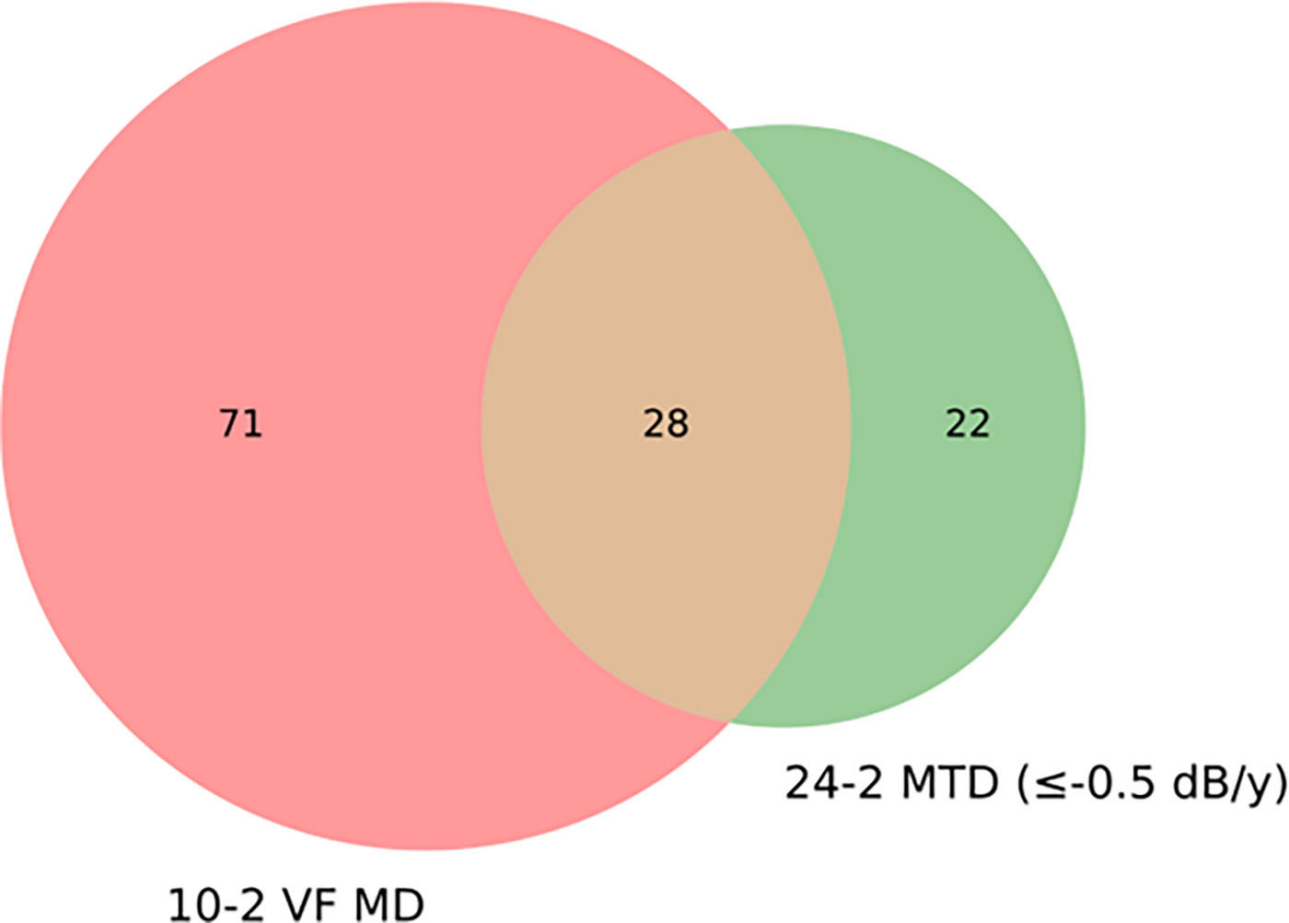
Venn diagram comparing progression classifications between MD_10_ and MTD_24_ (≤−0.5 dB/year). The *red circle* represents cases identified as progressing based on MD_10_, whereas the *green circle* represents those classified as progressing by MTD_24_. The overlapping region (*n* = 28 [9.9%]) indicates cases classified as progressing by both methods, whereas the non-overlapping areas represent cases uniquely identified by either metric (MD_10_: 71 [25.2%]; MTD_24_: 22 [7.8%]).

Table 1 summarizes the criteria for VF progression used in this study and the number of eyes meeting each criterion. The percentage of series identified as showing progression varied across methods. For the 10-2 tests, 56 (20.6%) eyes showed progression using PLR_10_ criterion, and 99 (35.1%) eyes showed progression based on MD_10_ (≤−0.7 dB/y) criteria. For the 24-2 tests, 50 (17.7%) eyes showed progression based on MTD_24_ using a threshold of ≤−0.5 dB/year, whereas only nine (3.2%) eyes met the stricter criterion of ≤−1.0 dB/year for MTD_24_. Additionally, 55 (19.5%) eyes were identified as progressing based on the MD_24_ (≤−0.5 dB/y) criterion, and 58 (20.6%) eyes showed progression using PLR_24_ method. The GPA indicated progression in 86 (30.5%) eyes, whereas the AGIS and CIGTS scores identified progression in 50 (17.7%) and 76 (27.0%) eyes, respectively. Figure 2 shows the Venn diagram illustrating the overlap between eyes classified as progressing by 10-2 VF MD and 24-2 MTD (≤−0.5 dB/y). The 10-2 VF MD method identified 71 eyes, whereas 24-2 MTD (≤-0.5 dB/y) identified 22, with both methods agreeing on 28 eyes.

**Figure 3.**
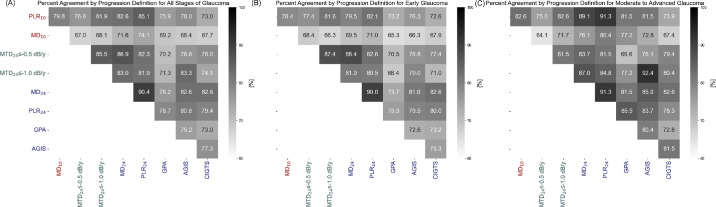
Percent agreement for 10-2/24-2 visual field progression definition. (**A**) all stages of glaucoma; (**B**) early glaucoma; (**C**) moderate to advanced glaucoma. *Darker blue shades* indicate higher agreement and *lighter green* to *yellow shades* indicate lower agreement.

Figure 3 illustrates the percentage agreement among different VF progression definitions for (A) all stages of glaucoma, (B) early glaucoma, and (C) moderate-to-advanced glaucoma. The percent agreement values between the 10-2 methods and the MTD_24_ methods ranged from 67.0% to 81.9%, whereas the agreement between the 10-2 and other 24-2 methods showed a range from 67.7% to 85.1% for all stages of glaucoma.

**Figure 4.**
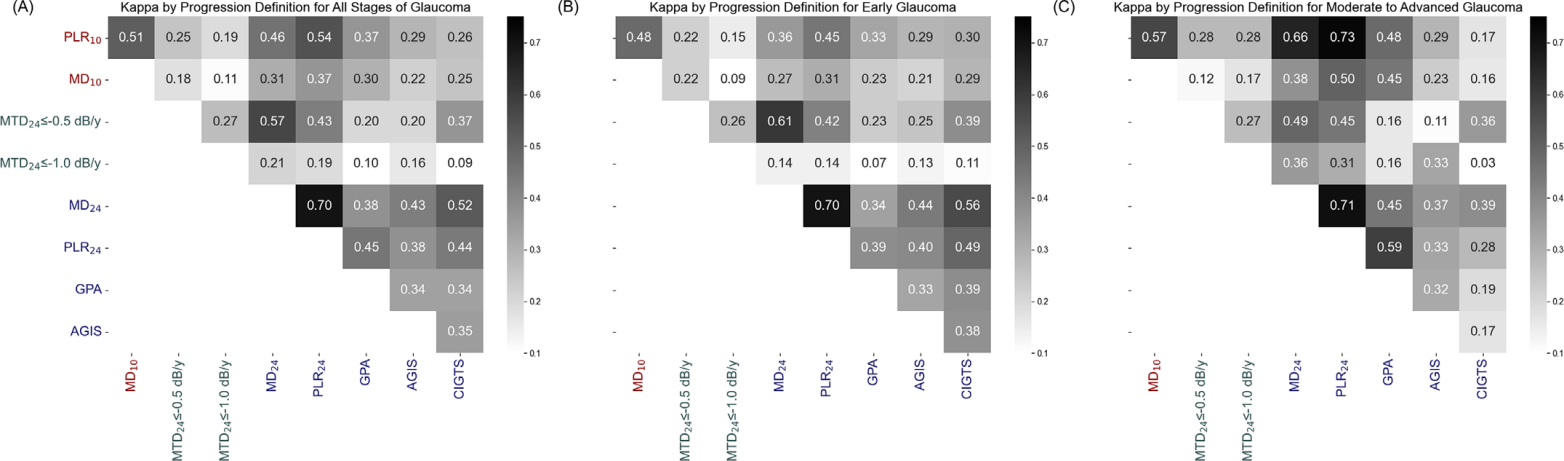
Kappa for 10-2/24-2 visual field progression definition. (**A**) all stages of glaucoma; (**B**) early glaucoma; (**C**) moderate to advanced glaucoma. Darker color indicates higher agreement.

Figure 4 shows the kappa values comparing the 10-2 methods with the MTD_24_ and other 24-2 methods. The agreement between the 10-2 methods and MTD_24_ methods (Fig. 4A) ranges from 0.11 to 0.25, whereas the kappa values between the 10-2 methods and other 24-2 methods range from 0.22 to 0.54. Kappa values between the 10-2 methods and MTD_24_ methods are lower in early glaucoma (0.09–0.22, Fig. 4B) and slightly higher in moderate-to-advanced glaucoma (0.12–0.28, Fig. 4C). The kappa values between the 10-2 and 24-2 methods are low in early glaucoma (0.21–0.45, Fig. 4B). In moderate-to-advanced glaucoma, agreement is higher with MD_24_, PLR_24_, and GPA (0.38–0.73, Fig. 4C) but remains low with AGIS and CIGTS (0.16–0.29, Fig. 4C).

**Figure 5.**
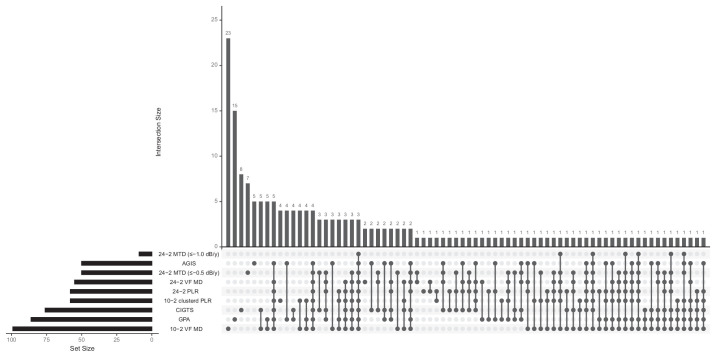
UpSet plot illustrates the classification of eye progression as determined by various methods. The horizontal histogram (*bottom left*) displays the total number of eyes identified as progressing by each method. The matrix (*bottom*) indicates the methods involved in each analysis, marked by *black dots*. Intersections of two or more methods are highlighted by *black dots* linked with a *continuous black line*. The vertical histogram shows the count of eyes that were determined to be progressing either by a single method or by intersecting methods. Out of the total, 103 eyes (36.5%) were not identified as progressing by any of the methods.

Figure 5 presents the UpSet plot showing VF progression according to different criteria and methods. The histogram displays the total number of eyes identified as progressing by each method. The variability in detection rates across different methods underscores the lack of agreement in identifying VF progression, with no single method consistently demonstrating superiority. Out of the total, 103 eyes (36.5%) were not identified as progressing by any of the methods. UpSet plot in Union mode, capturing all eyes identified as progressing by at least one method and illustrating the cumulative detection rate across different criteria is provided in Supplementary Figure S3.

**Figure 6.**
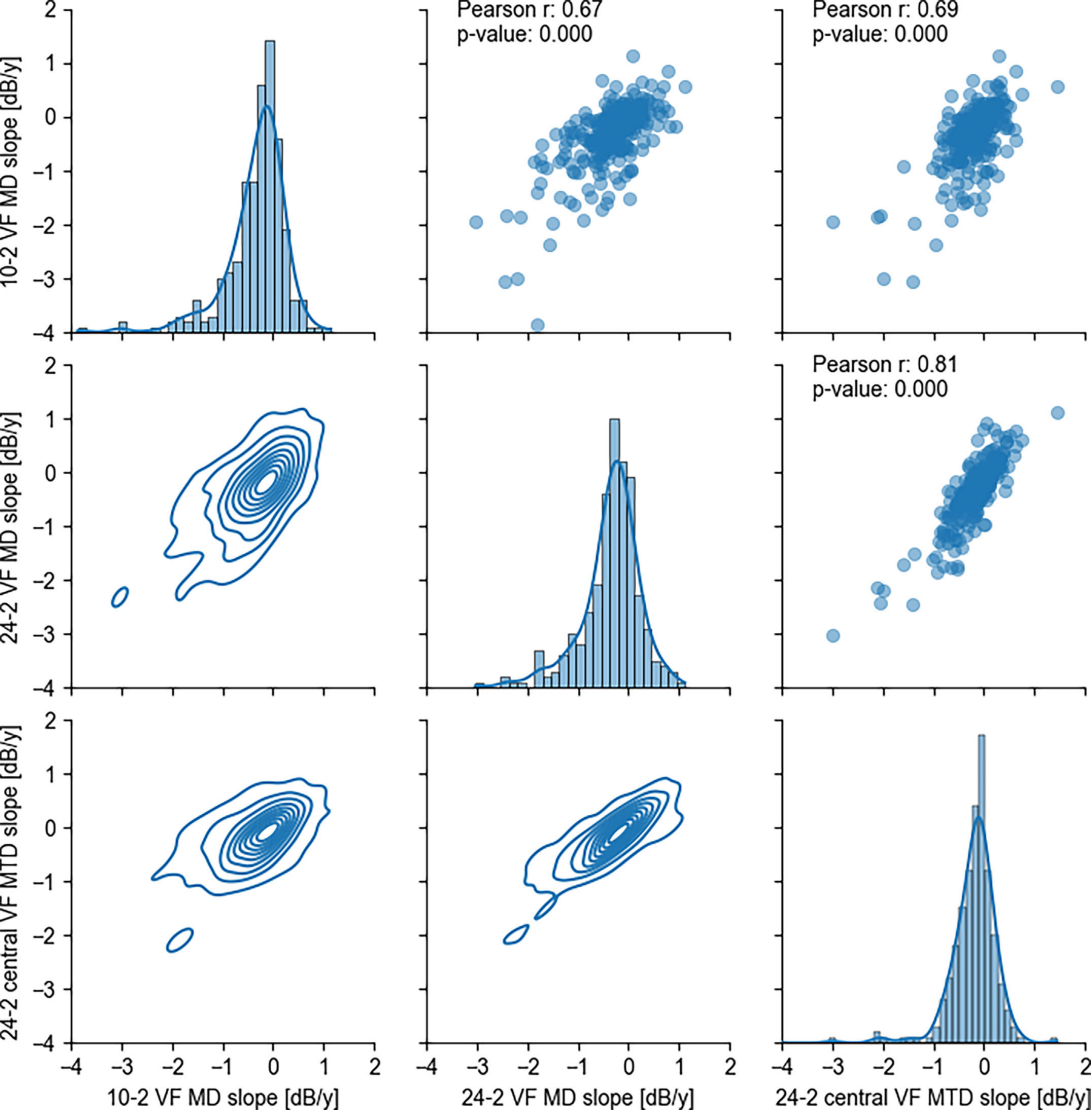
Scatter matrix among 24-2 VF MD slope, 10-2 VF MD slope, and 24-2 central VF MTD slope. This figure presents a scatter matrix illustrating the relationships among three indices: 24-2 VF MD slope, 10-2 VF MD slope, and 24-2 central VF MTD slope. Each panel in the *upper triangle* shows a scatter plot with corresponding Pearson correlation coefficients (*r*) and *P* values annotated in the *upper right corner*. The diagonal panels depict histograms with overlaid KDE lines, showing the distribution of individual indexes. Panels in the *lower triangle* display KDE plots, illustrating the density distribution of index pairs. KDE, kernel density estimate.

Figure 6 shows a scatter matrix illustrating the relationships among three indices: MD_24_ slope, VF_10_ MD slope, and MTD_24_ slope. The Pearson correlation coefficients indicate strong correlations between these indices, with values of 0.67 (MD_10_ slope and MD_24_ slope), 0.69 (MTD_24_ slope and MD_10_ slope), and 0.81 (MTD_24_ slope and MD_24_ slope), (all *P*s < 0.001).

**Figure 7.**
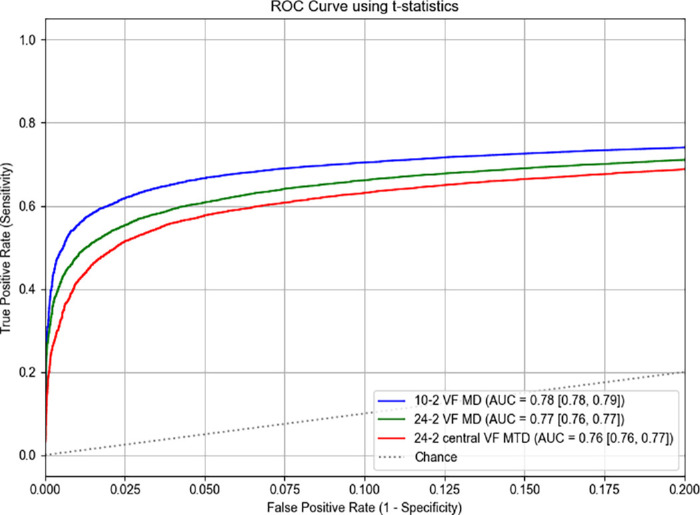
ROC curves using *t*-statistics derived from ordinary least squares regression for three summary VF metrics: 10-2 VF MD, 24-2 VF MD, and central 24-2 MTD.

**Figure 8.**
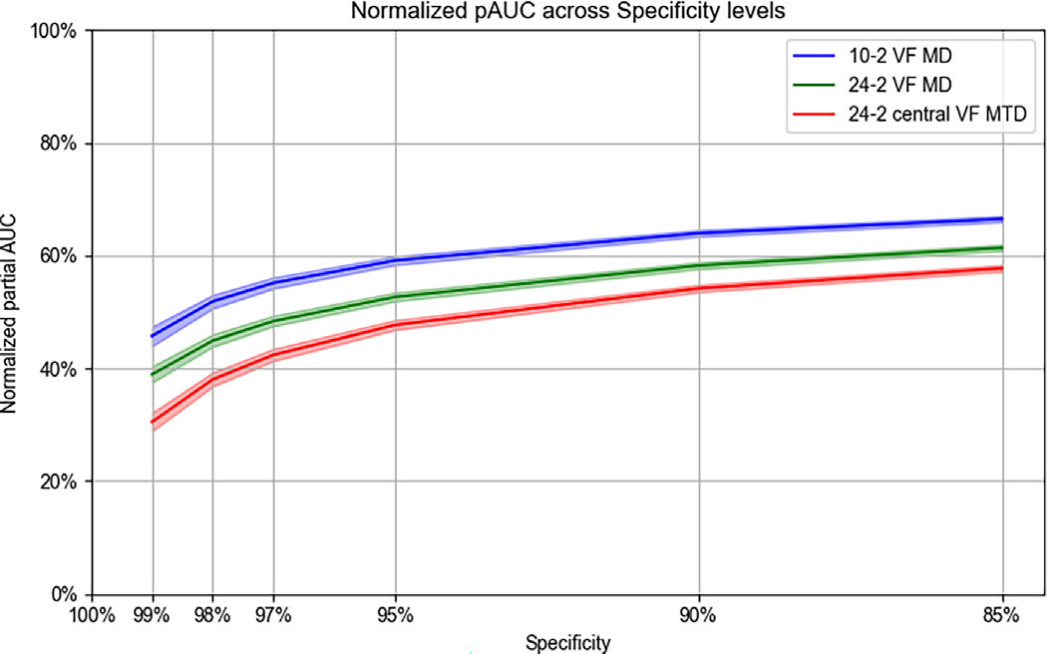
Normalized pAUC for each VF metric at different specificity rate thresholds. *Bars* indicate the mean pAUCs with 95% confidence intervals based on bootstrap replicates.

Figure 7 shows the ROC curve using *t*-statistics, restricted to the range of FPR 0–0.2, demonstrating that MD_10_ had highest AUC (0.78 [95% CI, 0.78–0.79]), compared to MD_24_ (0.77 [95% CI, 0.76–0.77]) and MTD_24_ (0.76 [95% CI, 0.76–0.77]) (*P* < 0.001, respectively). Figure 8 illustrates the normalized pAUCs at different FPR levels. At a FPR of 0.05 (i.e., 95% specificity), the normalized partial AUC was 0.59 (95% CI, 0.58–0.60) for MD_10_, 0.53 (95% CI, 0.52–0.53) for MD_24_, and 0.48 (95% CI, 0.47–0.49) for MTD_24_ (All between-group *P* values were <0.001). The values at other FPR are provided in Supplementary Table S1. Supplemental Figure S4 shows the distribution of *t*-statistics for simulation datasets.

## Discussion

This investigation aimed to assess the agreement of various criteria for evaluating VF progression in eyes with CVFD and found low to moderate agreement across the employed methodologies. Understanding these discrepancies is crucial for making informed decisions regarding patient management and treatment interventions. Different methods can lead to different interpretations of disease progression, affecting clinical outcomes and patient care strategies. In the present study, the proportion of eyes with detected central progression was higher for 10-2 VF compared to 24-2, suggesting that clinicians should not solely rely on the 24-2 VF assessment when monitoring glaucoma patients with central visual field involvement.

The use of different methods—including PLR_10_, MD_10_, MTD_24_, MD_24_, PLR_24_, GPA, AGIS scores, and CIGTS scores—demonstrated considerable differences in progression detection rates and agreement among methods. These findings align with earlier studies reporting variability in the detection of glaucomatous progression using different approaches.^10^^,^^11^^,^^15^^–^^17^^,^^27^^,^^28^ In this study, the mean rate of MD_24_ slope was −0.35 dB/year, whereas the mean rate for the MD_10_ slope was −0.33 dB/year, indicating similar overall rates of decline in retinal sensitivity across these two testing strategies. However, the proportion of series detected as progressing varied significantly among the methods, ranging from as low as 3.2% for MTD_24_ (≤−1.0 dB/year) to 30.5% for GPA. The rate of VF progression detection is influenced by factors such as the frequency of examinations, the duration of follow-up, baseline severity, and the specific criteria used, making study-by-study comparisons challenging.^11^ However, each criterion, excluding the most stringent, identified progression in approximately 20% to 30% of cases, which is consistent with findings from previous studies.^18^ The low agreement between criteria highlights a fundamental issue in VF progression detection. The absence of a universally accepted “gold standard” for monitoring VF progression in glaucoma further complicates clinical decision-making. Different methods often disagree, making it difficult to rely solely on one approach for accurate assessment.

In clinical trials, binary outcomes are frequently utilized, allowing for logistic regression, cox model, and other interpretations that are highly accessible to clinicians. Binary outcomes simplify the analysis and communication of results, making it easier to apply standardized statistical methods and generate clear, actionable insights. They are particularly useful in regulatory settings where clear, definitive endpoints are required for decision-making processes. Additionally, binary outcomes facilitate the comparison of results across different studies and can provide a clear threshold for clinical intervention, aiding clinicians in making timely decisions. However, capturing progression as a binary outcome can dilute the information, losing the rate of progression, which is crucial for making treatment decisions.^29^^,^^30^ This loss of information could explain the low agreement observed with binary outcomes and the higher agreement seen with continuous variables in our study. Binary outcomes tend to ignore the subtleties of gradual changes, leading to potential underestimation or overestimation of disease progression. The different methods showed a lack of agreement in identifying the same eyes as progressing, highlighting the limitations of binary assessments in clinical practice.

To improve the agreement of VF progression detection, hybrid outcome measures and advanced statistical methods can be employed. Combining both binary and continuous measures of VF progression can provide a more comprehensive assessment. Continuous measures can capture the rate of progression, while binary outcomes offer clear thresholds for clinical decision-making. This dual approach can mitigate the loss of detailed information that occurs with dichotomous outcomes. Developing composite endpoints that integrate multiple aspects of VF progression can offer a more holistic view of disease progression, enhancing sensitivity and specificity in detecting meaningful changes.^31^^,^^32^ Composite endpoints summarize different categories into a single endpoint, increasing the overall sensitivity to reach acceptable results (i.e., progression). When used in clinical trials, more sensitive composite endpoints can allow for smaller, shorter trials to be conducted without a loss in statistical power.^31^^,^^32^ This efficiency can facilitate faster and more effective evaluation of treatments. Furthermore, implementing machine learning algorithms to analyze VF data can identify subtle patterns and trends that may not be apparent with traditional statistical methods, enhancing the predictive power of VF progression models by leveraging large datasets and complex interactions among variables.^21^^,^^33^

Matching specificity is important when comparing progression detection methods, particularly when definitions rely on statistical thresholds. Whereas a *P* value < 0.05 in linear regression implies a 5% false-positive rate under ideal conditions, this assumption does not hold when progression is defined by slope thresholds or rule-based criteria. Specificity can vary between progression method,^17^ making direct comparisons problematic. To examine this issue, we constructed a simulation dataset assuming a slope of 0 dB/year. In trend-based analyses, progression is often defined by the *P* value from ordinary least-squares regression, using a threshold such as *P* < 0.05 to indicate statistically significant decline. However, this binary classification is sensitive to both sample size and variability, because the *P* value reflects a combination of the estimated slope and its standard error. The *t*-statistic, calculated as the slope divided by its standard error, serves as a continuous and standardized indicator of change. This makes it more suitable for comparing eyes with different levels of measurement noise. Although both the *P* value and *t*-statistic are calculated from the same regression model, the *t*-statistic offers a continuous measure of change that reflects both the estimated slope and the variability of the data. Unlike *P* values, which require an arbitrary cutoff to define progression, the t-statistic can be used directly to compare eyes across a wide range of signal-to-noise conditions. By treating the *t*-statistic as a continuous test variable, we were able to generate ROC curves that reflect performance across the entire range of potential thresholds, enabled comparison across different VF metrics while controlling for specificity.

To our knowledge, no previous study has directly compared progression detection between the 10-2 MD slope and cluster-based PLR criteria. In the present study, we adopted a relatively stringent definition for PLR, based on the methodology proposed by De Moraes et al.^12^ This conservative criterion—designed to minimize false-positive rates—may have contributed to the lower frequency of progression detection by PLR_10_ compared to MD_10_, which may be more sensitive to diffuse central loss. Because GPA is currently available only for the 24-2, 30-2, and 24-2C test patterns, its ability to detect progression involving the central 10° of the VF may be limited. Although the clinical utility of 10-2–based event analyses has been demonstrated in prior studies,^34^ such functionality is not currently available in commercial software. The lack of a standardized, vendor-supported progression algorithm for 10-2 testing represents an ongoing limitation in clinical practice.

This study has some limitations. The retrospective nature of the analysis, where not all eyes had VF tests performed at the same frequency, potentially affected progression detection. Furthermore, the study did not account for the potential impact of varying follow-up intervals on the detection rates. The lack of a consistent follow-up interval can introduce variability, impacting the overall assessment of VF progression. Another limitation is systematic lens opacity grading was not available during follow-up to directly investigate the possible impact of changes in lens opacity on VF results. Although eyes with non-glaucomatous VF defect were excluded, minor causes of VF loss, such as dry eye disease and mild cataracts, were not considered. Additionally, some patients (87 eyes [30.9%]) were pseudophakic at baseline, and 63 eyes (22.3%) underwent cataract surgery during the study. It is possible that cataract surgery may have affected the study results when comparing VF tests. These factors introduce potential variability that could impact the detection of VF progression and the interpretation of our findings.

## Conclusions

In conclusion, our study highlights the lack of a widely accepted methodology for monitoring VF progression in glaucoma patients with CVFD. The greater proportion of eyes showing central progression with the 10-2 VF test suggests that the 24-2 VF may underestimate central VF progression. The low to moderate agreement between the 24-2 and 10-2 VF tests underscores the limitations of relying solely on one approach, as the 24-2 VF may not adequately assess central VF progression. Further research is needed to develop and validate standardized criteria that can reliably detect central VF progression to improve patient outcomes in glaucoma management.

## Supplementary Material

Supplement 1
