# Supplementary material for: Comparison of Methods for Visual Field Progression in Eyes With Central Visual Field Defects
Source: Transl Vis Sci Technol. 2025 Nov 6;14(11):3. doi: 10.1167/tvst.14.11.3 (PMC12599513; doi:10.1167/tvst.14.11.3)
Supplement: Supplement 1 [file tvst-14-11-3_s001.docx]

Supplementary Figure 1. Visual field (VF) sectors of the 10-2 VF, segmented for clustered pointwise linear regression (PLR) analysis. The sector map is based on the framework proposed by Hood et al., which delineates five distinct VF zones according to their susceptibility to macular damage. Zone 1 = superior nasal (SN) zone; zone 2 = superior temporal (ST) zone; zone 3 = superior temporal band (STB) zone; zone 4 = inferior temporal (IT) zone; and zone 5 = inferior nasal (IN) zone.


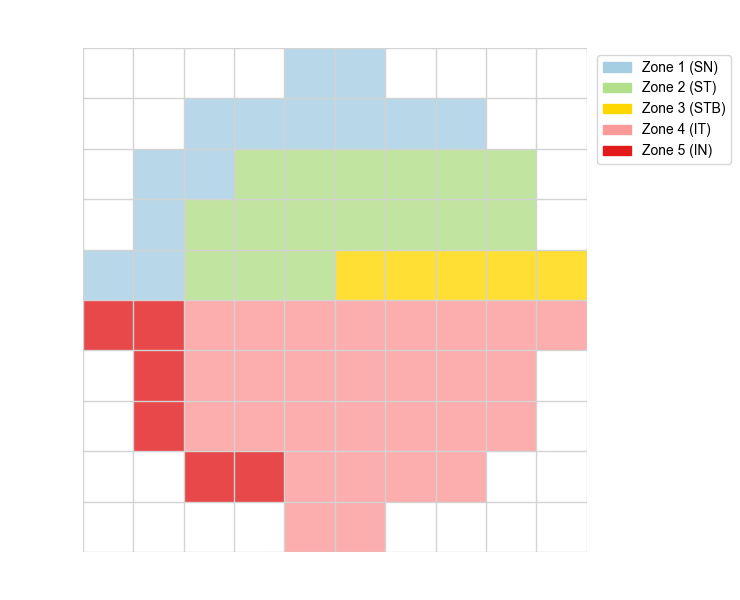


Reference: Hood DC, Raza AS, de Moraes CG, Liebmann JM, Ritch R. Glaucomatous damage of the macula. Prog Retin Eye Res. 2013 Jan;32:1-21.

Supplementary Figure 2. Agreement between clustered 10-2 PLR and 10-2 VF MD slopes. Since there is no established cutoff for the 10-2 VF MD slope, the value with the highest accuracy was determined based on the clustered PLR and MD slope. Abbreviations: MD = mean deviation; MTD = mean total deviation; PLR = pointwise linear regression; VF = visual field.


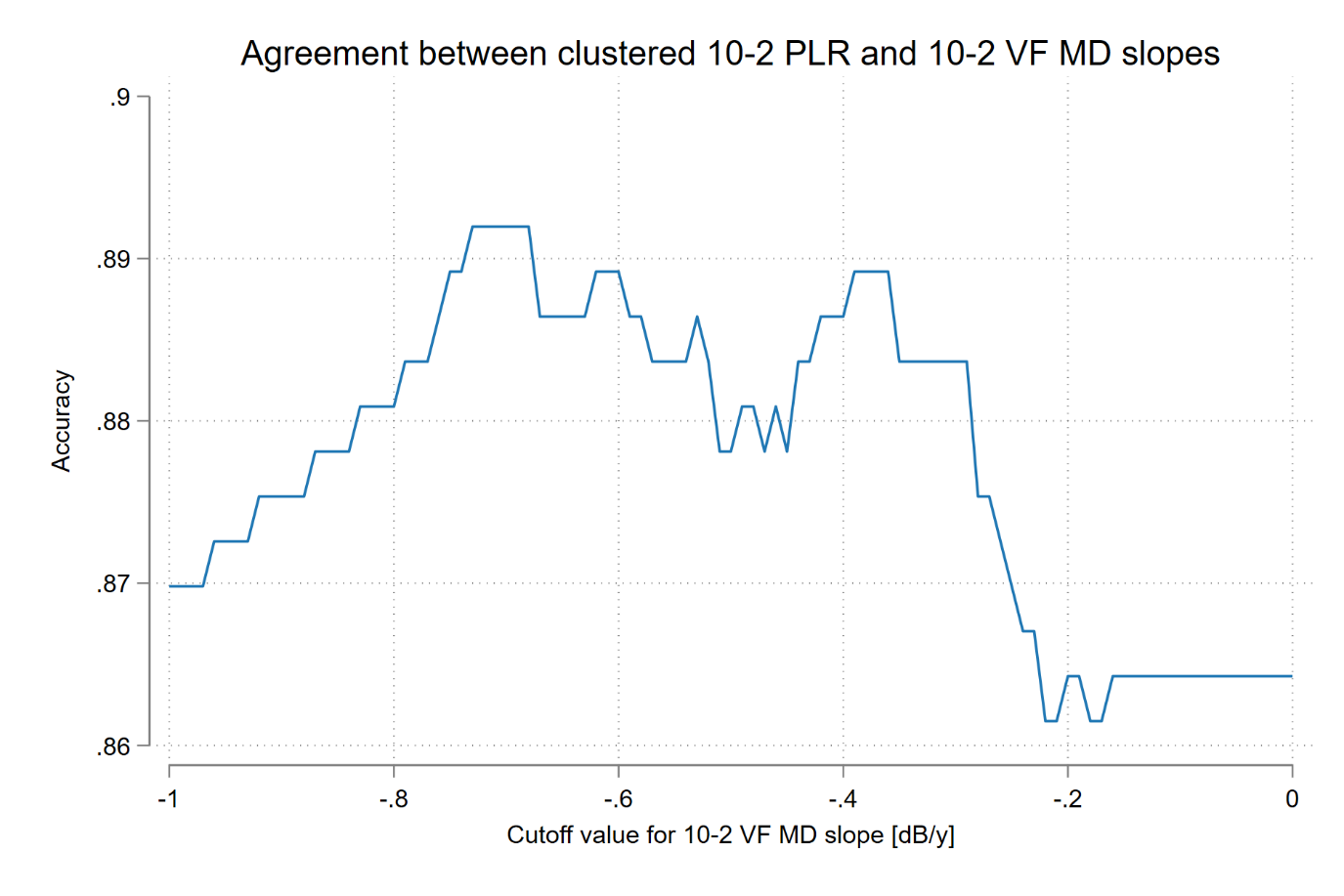


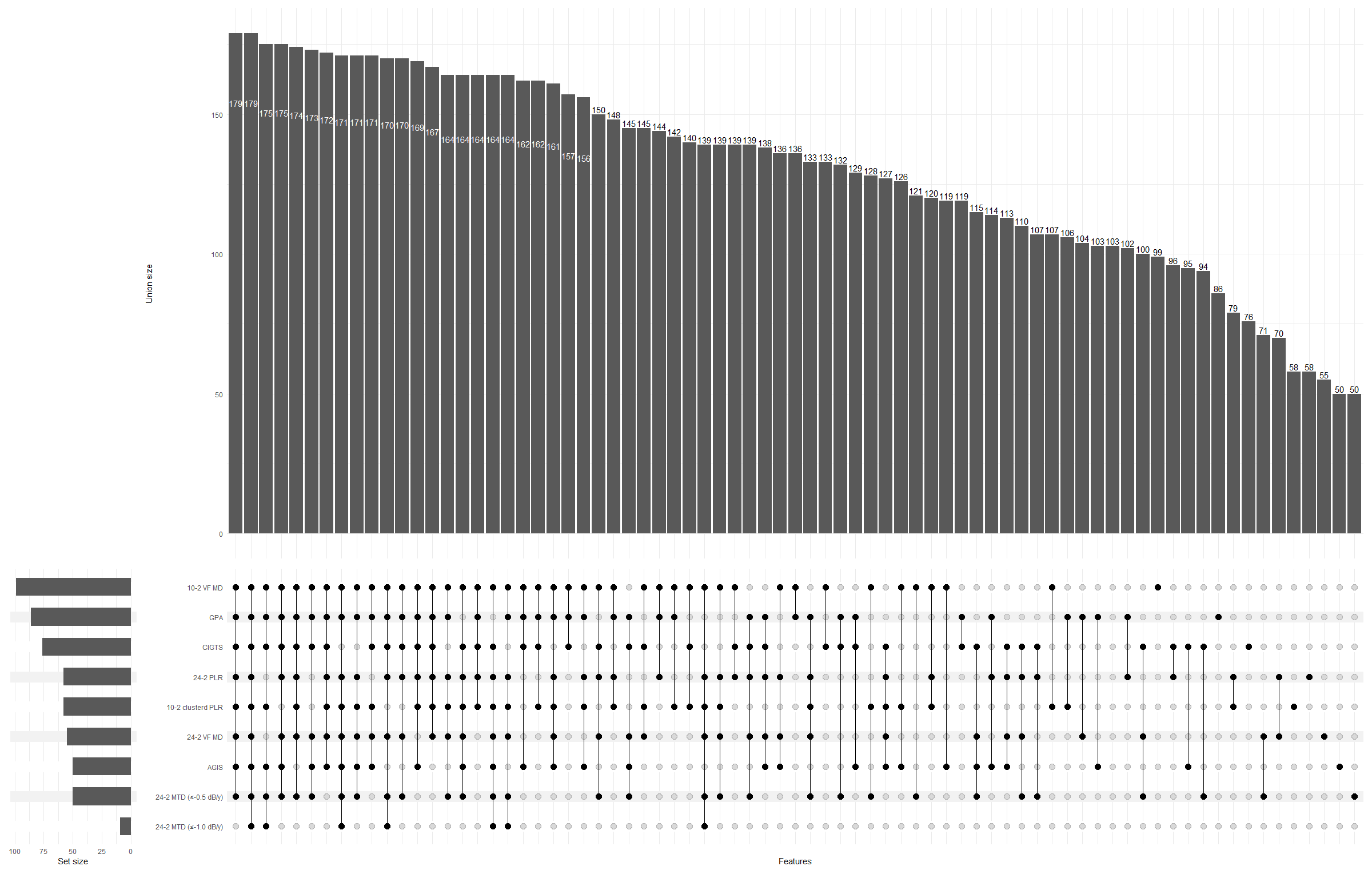
Supplementary Figure 3. The UpSet plot visualizes the classification of eye progression as determined by various methods in Union mode. The horizontal histogram (bottom left) represents the total number of eyes identified as progressing by each method. The matrix (bottom) indicates the combinations of methods contributing to each analysis, with black dots marking the presence of a method and connected black dots representing multiple methods contributing to the same classification. The vertical histogram (top) displays the union size, which accounts for the total number of eyes identified as progressing by at least one of the included methods. Unlike intersection-based analysis, Union mode considers all eyes that meet the criteria for any single method, leading to a broader representation of progression detection.

Supplementary Figure 4. Distribution of longitudinal visual field (VF) slopes for both simulation datasets. Histograms show the slope distributions of 10-2 VF mean deviation (MD), 24-2 VF MD, and central 24-2 mean total deviation (MTD). Each metric was derived from simulation data based either on observed longitudinal changes or a constant (zero-slope) baseline. The dataset with observed decline exhibits broader, left-shifted distributions,
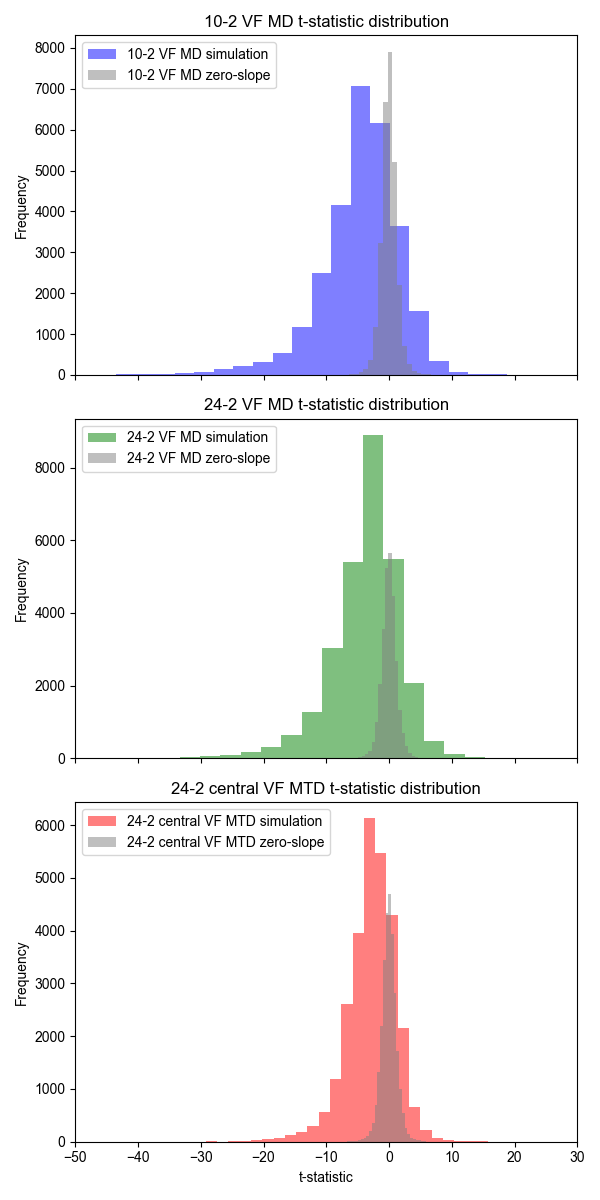
consistent with measurable VF deterioration.

Supplementary Table 1. partial area under the curves values at different false positive rates

| FPR value | 10-2 VF MD | 24-2 VF MD | 24-2 central VF MTD | P-value |
| --- | --- | --- | --- | --- |
| 0.05 | 0.59 (0.58, 0.60) | 0.53 (0.52, 0.53) | 0.48 (0.47, 0.49) | <0.001 for all between-group P values |
| 0.10 | 0.64 (0.63, 0.65) | 0.58 (0.58, 0.59) | 0.54 (0.53, 0.55) | <0.001 for all between-group P values |
| 0.15 | 0.67 (0.66, 0.67) | 0.61 (0.61, 0.62) | 0.58 (0.57, 0.58) | <0.001 for all between-group P values |
| 0.20 | 0.68 (0.68, 0.69) | 0.64 (0.63, 0.64) | 0.60 (0.60, 0.61) | <0.001 for all between-group P values |
| Abbreviations: FPR = false positive rate; MD = mean deviation; MTD = mean total deviation; VF = visual field. | | | | |
